# Construction and validation of a nutritional status (CONUT)-based nomogram for predicting prolonged hematological toxicity in relapsed/refractory multiple myeloma after CAR-T cell therapy

**DOI:** 10.3389/fnut.2026.1729151

**Published:** 2026-02-23

**Authors:** Peng Xu, Xin-Ying Duan, Qi-Wen Feng, Ya-Wen Wang, Yang Liu, Huan-Xin Zhang, Kun-Ming Qi, Zhen-Yu Li, Qing-Yun Wu

**Affiliations:** 1 Blood Disease Institute, Xuzhou Medical University, Xuzhou, Jiangsu, China; 2 Department of Hematology, The Affiliated Hospital of Xuzhou Medical University, Xuzhou, Jiangsu, China; 3 Jiangsu Key Laboratory of Bone Marrow Stem Cells, Xuzhou, Jiangsu, China

**Keywords:** CAR-T cell therapy, CONUT score, multiple myeloma, nomogram, prolonged hematological toxicity

## Abstract

**Background aim:**

Chimeric antigen receptor (CAR)-T cell therapy is highly effective for relapsed/refractory multiple myeloma (R/R MM). Prolonged hematological toxicity (PHT) is a significant adverse event that adversely affects patient outcomes; however, specific predictive tools are lacking. Our prior study demonstrated that baseline Controlling Nutritional Status (CONUT) affects the prognosis of R/R MM patients receiving CAR-T cell therapy. We aimed to develop and validate a nomogram based on CONUT score for the early prediction of PHT after CAR-T cell therapy.

**Methods:**

This retrospective study included 302 consecutive patients with R/R MM who received CAR-T cell therapy. Patients were randomly allocated to training and validation cohorts (7:3 ratio). The primary endpoint was prolonged grade 3/4 neutropenia >28 days; predictors were identified using logistic regression. The model's performance was assessed by the area under the curve (AUC), calibration curves, and decision curve analysis (DCA).

**Results:**

Multivariable analysis confirmed four independent predictors for the primary endpoint (prolonged grade 3/4 neutropenia >28 days): high tumor burden (*p* = 0.013), ferritin (*p* = 0.002), interferon-γ (IFN-γ, *p* = 0.018), and CONUT score (*p* = 0.011). The nomogram built on these factors demonstrated a bias-corrected AUC of 0.815 in the training cohort, which was superior to the CAR-HEMATOTOX model (AUC: 0.706, *p* < 0.001). The predictive performance remained robust in the internal validation cohort (AUC: 0.824). The calibration curves showed good agreement between prediction and observation, and DCA confirmed the clinical utility of the model. The nomogram also exhibited excellent discriminative ability for predicting a composite PHT endpoint (AUC: 0.821, *p* = 0.417).

**Conclusion:**

We developed a validated nomogram that incorporates the baseline CONUT score and key clinical variables (e.g., tumor burden, ferritin, IFN-γ) to effectively predict PHT risk in R/R MM patients after CAR-T cell therapy, thereby facilitating early risk stratification and guiding personalized management.

## Introduction

Multiple myeloma (MM) is the second most common hematologic malignancy, caused by the malignant proliferation of plasma cells in the bone marrow (1, 2). Currently, clinical treatment modalities for MM include chemotherapy, autologous hematopoietic stem cell transplantation (Auto-HSCT), and targeted agents such as proteasome inhibitors, immunomodulatory drugs, and monoclonal antibodies; however, patients eventually develop disease recurrence (3–5). Therefore, treating relapsed/refractory (R/R) MM remains a major clinical challenge. Chimeric antigen receptor (CAR)-T cell therapy has emerged as a promising immunotherapeutic strategy. Growing evidence from clinical studies confirms its remarkable efficacy in R/R MM, substantially improving patient prognosis (6–9).

Despite its therapeutic benefits, CAR-T cell therapy is associated with unique toxicities, most notably cytokine release syndrome (CRS) and immune effector cell-associated neurotoxicity syndrome (ICANS) (10, 11). In contrast, hematological toxicity (HT) has received comparatively less attention. Notably, HT not only poses a substantial healthcare burden but also adversely affects patient outcomes. For instance, a retrospective study by the Transplant Complications Working Party of the EBMT demonstrated that while severe cytopenia did not impact overall survival (OS), it was associated with significantly shorter progression-free survival (PFS) after CAR-T therapy (12). Similarly, Li et al. (13) reported that R/R MM patients after CAR-T therapy with prolonged HT (PHT) had significantly shorter median PFS and OS compared to those without PHT and identified PHT as an independent risk factor for both endpoints. The current tools for assessing HT, such as the CTCAE v5.0 and the CAR-HEMATOTOX model, have limitations: CTCAE v5.0 focuses primarily on cytopenia severity, while the CAR-HEMATOTOX model shows relatively low specificity (14). Therefore, there is an urgent need to develop a more specific predictive model for CAR-T-related HT in R/R MM to facilitate early intervention.

In recent years, the predictive role of nutritional status in treatment response and survival prognosis across various malignant tumors has garnered increasing attention (15–17). Among nutritional assessment tools, the Controlling Nutritional Status (CONUT) score—an nutritional-immunological assessment index based on serum albumin levels, peripheral blood lymphocyte counts, and total cholesterol levels—is not only simple to calculate but also has demonstrated robust predictive value in several hematological malignancies (18, 19). Our center's previous study demonstrated that R/R MM patients with better nutritional status (low CONUT score group) prior to CAR-T cell therapy exhibit faster recovery of complete blood cells and superior PFS and OS after CAR-T cell therapy (20). However, studies on the association between CONUT score and PHT after adoptive cell therapy (ACT) remain scarce.

Therefore, we conducted a retrospective study to identify independent risk factors for PHT in patients with R/R MM after CAR-T therapy, incorporating baseline CONUT scores and other clinical indicators. Based on these factors, we developed and validated a nomogram for the early prediction of PHT, with the goal of enabling accurate risk stratification and informing targeted preventive strategies.

## Patients and methods

### Study population

**Figure 1:**
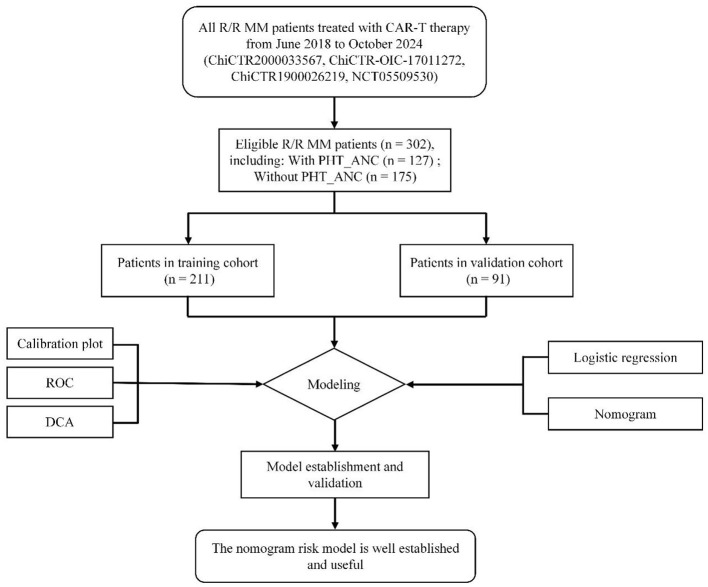
Flowchart of the PHT_ANC risk modeling process for R/R MM patients treated with CAR-T therapy. ROC, receiver operating characteristic; DCA, decision curve analysis.

We conducted a single-center retrospective study of 302 patients with R/R MM treated with CAR-T therapy at the Affiliated Hospital of Xuzhou Medical University from June 2018 to October 2024 (Figure 1). The administered CAR-T regimens included: (1) monospecific BCMA-targeted CAR-T cells, (2) monospecific GPRC5D-targeted CAR-T cells, (3) BCMA/CD19 bispecific CAR-T cells, or (4) BCMA/GPRC5D bispecific CAR-T cells. The study was approved by the hospital's Ethics Committee and conducted in accordance with the Declaration of Helsinki. This retrospective analysis utilized data from patients at our center who were enrolled in prospective CAR-T clinical trials registered on Chictr.org.cn (ChiCTR2000033567, ChiCTR-OIC-17011272, ChiCTR1900026219) and ClinicalTrials.gov [NCT05509530; a phase 1 trial of anti-BCMA/GPRC5D bispecific CAR-T cells (8)]. All participants provided written informed consent for the original trials, which included authorization for subsequent data analysis. Patient inclusion and exclusion criteria were consistent with previously published studies (8, 21). Prior to CAR-T infusion, all patients underwent lymphodepletion with fludarabine (30 mg/m^2^ per day on days −5 to −3) and cyclophosphamide (750 mg/m^2^ on day −5).

### Data collection and definitions

Based on clinical experience, baseline demographic and clinicopathological variables were collected for patients with R/R MM prior to lymphodepletion. The variables considered for analysis included age, gender, myeloma subtype, prior treatment history, tumor burden, cytogenetic abnormalities, extramedullary disease status, body mass index (BMI) and the following laboratory values: absolute neutrophil count (ANC), hemoglobin (Hb), platelets (PLT), ferritin, C-reactive protein (CRP), interleukin-6 (IL-6), interleukin-8 (IL-8), interleukin-10 (IL-10), interferon-α (IFN-α), interferon-γ (IFN-γ), and lactate dehydrogenase (LDH). The CONUT score was calculated from serum albumin, serum cholesterol, and total peripheral lymphocyte count. Patients were stratified into one of four CONUT score groups, as defined in Table S1.

Cytopenia was graded according to the Common Terminology Criteria for Adverse Events (CTCAE) version 5.0 (22). PHT was defined as grade 3 or 4 cytopenia persisting beyond day 28 (D28+). We defined two specific endpoints: PHT_ANC (grade 3/4 neutropenia lasting > 28 days) and a PHT_Composite (grade 3/4 neutropenia, anemia, or thrombocytopenia lasting > 28 days). CAR-HEMATOTOX score was calculated prior to lymphodepletion using the online CAR-HEMATOTOX calculator from the German Lymphoma Alliance (GLA) (14).

All data were retrospectively extracted from the electronic medical record system of the Affiliated Hospital of Xuzhou Medical University between June 2018 and October 2024. For each included patient, the collected dataset comprised baseline demographic, disease history, pre-lymphodepletion laboratory results, CAR-T product details, and post-infusion toxicity records. All baseline variables were obtained prior to lymphodepletion and were available for the entire cohort. To ensure accuracy, data extraction was performed independently by two investigators, with any discrepancies resolved through consensus.

### Statistical analysis and nomogram construction

Continuous variables are presented as mean ± standard deviation (SD) or median with interquartile range (IQR), depending on their normality distribution, and were compared using independent samples *t*-tests or non-parametric tests, respectively. Categorical variables are expressed as frequencies and percentages (*n*, %) and were compared using the Chi-square test. In this retrospective cohort, patients were randomly split into a training cohort and an internal validation cohort (7:3). Within the training cohort, univariate logistic regression was first used to identify baseline variables and CONUT scores associated with PHT_ANC. Variables significant at the *p* < 0.05 level were subsequently entered into a stepwise multivariate logistic regression model. The final multivariate model served as the basis for constructing a predictive nomogram for PHT_ANC.

The discriminatory capability of the nomogram for predicting PHT_ANC after CAR-T therapy was evaluated employing bootstrap validation. This process involved 200 repetitions of random sampling with replacement from the training set, with the mean area under the receiver operating characteristic curve (AUC) and its 95% confidence interval (CI) subsequently derived and compared to those of other predictors. The agreement between predictions and observations (calibration) was analyzed using a calibration curve constructed from 1,000 bootstrap resamples. Decision curve analysis (DCA) was utilized to quantify the clinical utility by estimating the net benefit across different threshold probabilities. To further assess the model's stability, the nomogram was deployed on the validation cohort, and its effectiveness was verified through the AUC, calibration curve, and DCA. The entire statistical analysis was performed using R software (version 4.3.2): R Foundation for Statistical Computing, Vienna, Austria.

## Results

### Clinical characteristics of patients

A total of 302 patients with R/R MM were included in this study and randomly assigned to a training cohort (*n* = 211, 69.9%) and a validation cohort (*n* = 91, 30.1%) at a 7:3 ratio. In the training cohort, the median age was 57 years (range: 34–73), 113 patients (53.6%) were male, and 123 patients (58.3%) were classified as R-ISS stage III. High tumor burden was present in 26.5% (*n* = 56), high-risk cytogenetic features in 25.6% (*n* = 54), and extramedullary lesions in 36.5% (*n* = 77). The median number of previous therapy lines was 4 (range: 2–14), and a history of HCT was reported in 63 patients (29.9%). The IgG M-protein subtype was the most frequent (64.5%, *n* = 136). Regarding CAR-T cell therapy, 49 patients (23.2%) received BCMA, 54 patients (25.6%) received GPRC5D, 87 patients (41.2%) received BCMA+CD19, and 21 patients (9.9%) received BCMA+GPRC5D. The incidence of PHT_ANC after CAR-T therapy was 45.9% (*n* = 97) in this cohort. This incidence is consistent with recent clinical studies reporting PHT_ANC rates (40%−50%) in R/R MM patients after CAR-T therapy (13, 23), verifying the representativeness of the study sample.

No statistically significant differences were observed between the two cohorts across all variables (all *p* > 0.05), indicating that the random allocation effectively balanced confounding factors. Detailed baseline clinical characteristics of both the training and validation cohorts are summarized in Table 1.

**Table 1:** Demographic and clinicopathological characteristics of the training and validation cohorts.

**Variables**	**Overall (*n* = 302)**	**Training cohort (*n* = 211)**	**Validation cohort (*n* = 91)**	***p*-value**
Age (years)	57 (34–76)	57 (34–73)	56 (39–76)	0.816
Gender, male	166 (54.9)	113 (53.6)	53 (58.2)	0.52
R-ISS stage III	179 (59.3)	123 (58.3)	56 (61.5)	0.612
Type of myeloma, IgG	185 (61.3)	136 (64.5)	49 (53.8)	0.095
**Type of CAR-T cell therapy**			
BCMA	64 (21.2)	49 (23.2)	15 (16.5)	0.189
GPRC5D	71 (23.5)	54 (25.6)	17 (18.7)	
BCMA+CD19	135 (44.7)	87 (41.2)	48 (52.7)	
BCMA+GPRC5D	32 (10.6)	21 (9.9)	11 (12.1)	
High tumor burden^a^	87 (28.8)	56 (26.5)	31 (34.1)	0.213
High-risk cytogenetic features^b^	73 (24.2)	54 (25.6)	19 (20.9)	0.464
Extramedullary lesions^c^	107 (35.4)	77 (36.5)	30 (32.9)	0.601
Previous HCT	99 (32.8)	63 (29.9)	36 (39.6)	0.109
Previous therapy lines	4 (2–14)	4 (2–14)	4 (2–10)	0.719
Neutrophil count, × 10^9^/L	2.1 (1.3–3.9)	2.3 (1.5–3.8)	2.2 (1.2–3.1)	0.425
Hemoglobin, g/L	95 (83–109)	93 (87–112)	96 (88–118)	0.54
Platelet count, × 10^9^/L	109 (76–154)	106 (73–146)	110 (75–152)	0.082
Ferritin, ng/ml	362.8 (218.2–819.3)	353.2 (216–805.1)	349 (221.1–796)	0.109
CRP, mg/L	5 (0.6–10)	5 (0.6–10)	5 (0.8–9)	0.315
IL-6, pg/ml	7 (2.8–12)	7 (5–10)	6 (3.1–8)	0.207
IL-8, pg/ml	5.2 (2.2–19.5)	5 (1.98–21.3)	5.4 (1.79–18.9)	0.481
IL-10, pg/ml	3.1 (1.8–6)	2.9 (1.6–5.7)	3.4 (1.3–5.4)	0.282
INF-α, pg/ml	4 (1.5–8.9)	4.3 (1.2–10)	4.1 (1.6–9.5)	0.724
INF-γ, pg/ml	7.3 (3.2–23.1)	7.5 (3.7–22.5)	7.2 (3.5–20.6)	0.314
LDH, U/L	205 (192–351)	198 (182–347)	187 (172–358)	0.207
BMI, kg/m^2^	22.5 (19.3–28.3)	22.5 (19–28.1)	23.1 (19.3–27.8)	0.38
CONUT score	4 (2–6)	4 (2–6)	4 (2–6)	0.417

Characteristics are summarized as median (interquartile ranges) or frequency (%).

^a^High tumor burden: defined as ≥50% clonal plasma cells or bone marrow plasma cells.

^b^High-risk cytogenetics was reported by investigators based on fluorescence *in situ* hybridization. A high-risk cytogenetic profile was defined by the presence of the following abnormalities: del(17p), t (4;14), or t (14; 16).

^c^Extramedullary diseases included tissue masses in extraosseous locations and bone-related plasmacytomas.

### Independent predictive factors in the training cohort

To identify factors associated with PHT_ANC in patients with R/R MM after CAR-T therapy, we first performed univariate logistic regression analysis, with PHT_ANC treated as a binary endpoint. Results showed that seven variables significantly correlated with PHT_ANC (all *p* < 0.05): high tumor burden (*p* = 0.013), platelet count (*p* = 0.032), serum ferritin (*p* = 0.002), CRP (*p* = 0.025), IL-6 (*p* = 0.015), IFN-γ (*p* = 0.018), and CONUT (*p* = 0.011). These findings offered initial evidence supporting the potential utility of these clinical and laboratory indicators for predicting PHT_ANC in this patient population.

Notably, univariate analysis only captures the unadjusted association between individual variables and PHT_ANC, as it fails to account for confounding among variables. To address this limitation and ensure methodological consistency, we included all statistically significant variables from the univariate analysis in a multivariate logistic regression model. After adjusting for confounding effects, the multivariate analysis confirmed four variables as independent risk factors for PHT_ANC in R/R MM patients after CAR-T therapy: high tumor burden (*p* = 0.017), serum ferritin (*p* = 0.024), IFN-γ (*p* = 0.028), and CONUT score (*p* = 0.012; Table 2). This result underscored that these four factors exert a robust, independent effect on PHT_ANC, even after controlling for the influence of other related variables.

**Table 2:** Univariate and multivariate analyses of the risk factors for PHT_ANC.

**Variables**	**PHT_ANC (*n* = 97)**	**Non-PHT_ANC (*n* = 114)**	**OR (univariable)**	**OR (multivariable)**
Age (years)	60 (42–74)	58 (37–75)	1.08 (0.67–1.13, *p* = 0.103)	
Gender, male	53 (54.6)	60 (52.6)	2.05 (0.84–3.16, *p* = 0.39)	
R-ISS stage III	57 (58.8)	66 (57.9)	2.37 (0.41–4.15, *p* = 0.218)	
Type of myeloma, IgG	64 (65.9)	72 (63.2)	1.16 (0.73–1.93, *p* = 0.419)	
**Type of CAR-T cell therapy**			
BCMA (reference)	21 (21.6)	28 (24.6)		
GPRC5D	24 (24.7)	30 (26.3)	1.13 (0.65–1.93, *p* = 0.209)	
BCMA+CD19	36 (37.1)	51 (44.7)	1.67 (0.49–2.17, *p* = 0.452)	
BCMA+GPRC5D	16 (16.5)	5 (4.4)	1.89 (0.77–2.35, *p* = 0.503)	
High tumor burden^a^	31 (31.9)	25 (21.9)	**2.21 (1.09–3.76**, ***p*** **=** **0.013)**	**1.96 (1.12–2.83**, ***p*** **=** **0.017)**
High-risk cytogenetic features^b^	28 (28.9)	26 (22.8)	2.18 (0.67–3.98, *p* = 0.65)	
Extramedullary lesions^c^	40 (41.2)	37 (32.5)	1.33 (0.43–2.54, *p* = 0.288)	
Previous HCT	27 (27.8)	36 (31.6)	0.86 (0.52–1.94, *p* = 0.522)	
Previous therapy lines	4 (2–14)	4 (2–10)	1.28 (0.66–1.53, *p* = 0.591)	
Neutrophil count, × 10^9^/L	1.8 (1.3–3.2)	2.1 (1.2–3.1)	1.16 (0.84–1.33, *p* = 0.098)	
Hemoglobin, g/L	91 (85–110)	94 (83–112)	1.29 (0.75–1.69, *p* = 0.106)	
Platelet count, × 10^9^/L	95 (69–136)	106 (75–142)	**1.09 (1.03–1.87**, ***p*** **=** **0.032)**	1.21 (0.89–1.94, *p* = 0.215)
Ferritin, ng/ml	350.9 (225.6–887)	318 (191.6–798)	**1.67 (1.25–4.15**, ***p*** **=** **0.002)**	**1.67 (1.15–3.28**, ***p*** **=** **0.024)**
CRP, mg/L	5 (2.4–12)	4 (0.6–9)	**1.58 (1.26–2.65**, ***p*** **=** **0.025)**	1.44 (0.79–2.09, *p* = 0.35)
IL-6, pg/ml	9 (5.3–23)	7 (2.8–10)	**1.38 (1.04–2.13**, ***p*** **=** **0.015)**	1.33 (0.87–2.03, *p* = 0.452)
IL-8, pg/ml	5.8 (2.3–29.3)	5.9 (1.78–22.9)	1.29 (0.67–1.25, *p* = 0.511)	
IL-10, pg/ml	3.2 (1.8–7.7)	2.9 (1.6–5.9)	2.06 (0.54–3.32, *p* = 0.214)	
INF-α, pg/ml	4.5 (1.4–12)	4.2 (1.6–10.2)	1.98 (0.55–2.61, *p* = 0.332)	
INF-γ, pg/ml	11.5 (7.7–42.5)	5.8 (3.5–20.4)	**1.48 (1.21–1.88**, ***p*** **=** **0.018)**	**1.27 (1.12–1.99**, ***p*** **=** **0.028)**
LDH, U/L	195 (180–337)	198 (178–347)	2.81 (0.511–3.19, *p* = 0.202)	
BMI, kg/m^2^	21.9 (18.8–28.1)	22.4 (18.3–29.8)	1.31 (0.84–2.93, *p* = 0.219)	
CONUT score	5 (3–7)	3 (2–6)	**2.12 (1.47–2.87**, ***p*** **=** **0.011)**	**2.09 (1.32–3.75**, ***p*** **=** **0.012)**

Characteristics are summarized as median (interquartile ranges) or frequency (%).

^a^High tumor burden: defined as ≥50% clonal plasma cells or bone marrow plasma cells.

^b^High-risk cytogenetics was reported by investigators based on fluorescence *in situ* hybridization. A high-risk cytogenetic profile was defined by the presence of the following abnormalities: del(17p), t (4;14), or t (14; 16).

^c^Extramedullary diseases included tissue masses in extraosseous locations and bone-related plasmacytomas.

Bold type highlights statistically significant results (defined as *p* < 0.05 using the Wald test).

### Predictive nomogram for PHT_ANC

**Figure 2:**
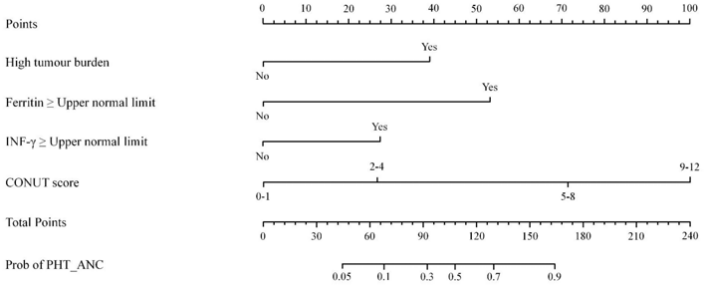
Characteristics in the nomogram to predict probability of occurrence of PHT_ANC after CAR-T cell infusion in R/R MM patients.

Subsequently, we developed a predictive nomogram to quantify the risk of PHT_ANC in R/R MM patients after CAR-T therapy, using the four independent risk factors. The nomogram was constructed based on the regression coefficients (β-values) of the multivariate logistic regression model: each independent risk factor was assigned a weighted score proportional to its β-value (higher β-values indicate greater predictive importance), and these scores were visually displayed on a dedicated “score axis” for intuitive interpretation (Figure 2).

In clinical practice, this nomogram enables personalized PHT_ANC risk assessment through three straightforward steps: (1) Map a patient's baseline values (e.g., serum ferritin level, CONUT score) to the corresponding score on each factor's scale; (2) Sum these individual scores to calculate a “total risk score”; (3) Convert the total risk score to a predicted probability of PHT_ANC via the nomogram's “risk axis.”

Eliminating the need to interpret complex raw regression outputs, this visual, user-friendly design makes PHT_ANC risk prediction more actionable in routine clinical practice.

### Nomogram validation

In the training cohort, the bias-corrected area under the curve (AUC) derived from Bootstrap validation was 0.815 (95% CI: 0.809–0.822), which exceeded the AUC values of other indicators: CAR-HEMATOTOX (AUC: 0.706, 95% CI: 0.702–0.713), high tumor burden (AUC: 0.617, 95% CI: 0.608–0.619), ferritin (AUC: 0.672, 95% CI: 0.669–0.677), IFN-γ (AUC: 0.641, 95% CI: 0.638–0.642), and CONUT score (AUC: 0.692, 95% CI: 0.688–0.701). All pairwise comparisons yielded a *p*-value < 0.001 (Table 3). Additionally, we utilized the established nomogram to predict PHT_Composite in patients with R/R MM after CAR-T therapy. Application of the nomogram to this patient cohort yielded an AUC of 0.821 (95% CI: 0.811–0.826) for PHT_Composite prediction, with a non-significant *p*-value of 0.417, validating its excellent discriminative performance. Notably, the AUC remained stable in the validation cohort, reaching 0.824 (95% CI: 0.813–0.831).

**Table 3:** AUC for the nomogram and model variables in the training and validation cohorts.

**Model variables**	Training cohort		Validation cohort	
	**AUC (95% CI)**	***p*** **-value**	**AUC (95% CI)**	***p*** **-value**
Nomogram for PHT_ANC	0.815 (0.809–0.822)		0.824 (0.813–0.831)	
Nomogram for PHT_Composite	0.821 (0.811–0.826)	0.417	0.842 (0.838–0.855)	0.564
CAR-HEMATOTOX (PHT_ANC)	0.706 (0.702–0.713)	**< 0.001**	0.711 (0.705–0.716)	**< 0.001**
High tumor burden (PHT_ANC)	0.617 (0.608–0.619)	**< 0.001**	0.622 (0.618–0.626)	**< 0.001**
Ferritin (PHT_ANC)	0.672 (0.669–0.677)	**< 0.001**	0.681 (0.679–0.684)	**< 0.001**
INF-γ (PHT_ANC)	0.641 (0.638–0.642)	**< 0.001**	0.652 (0.645–0.661)	**< 0.001**
CONUT score (PHT_ANC)	0.692 (0.688–0.701)	**< 0.001**	0.685 (0.681–0.98)	**< 0.001**

Bold type highlights statistically significant results (defined as *p* < 0.05 using the Wald test).

**Figure 3:**
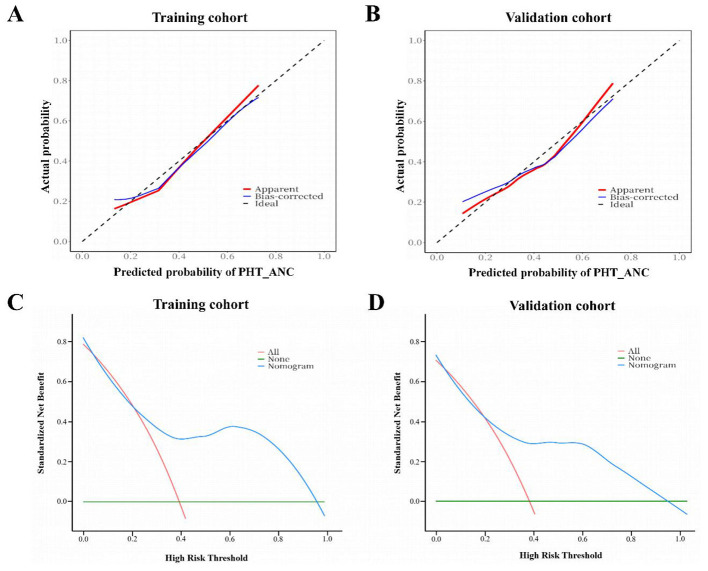
Calibration curves of the PHT_ANC risk nomogram in the training cohort **(A)** and validation cohort **(B)**. DCA curves of the PHT_ANC risk nomogram in the training cohort **(C)** and validation cohort **(D)**.

In both the training and validation cohorts, the (actual and calibrated) calibration curves closely overlapped with the ideal line, indicating that the nomogram's predicted outcomes were in good agreement with the observed real-world results (Figures 3A, B). DCA results depicted in Figures 3C, D demonstrate that within a relatively wide range of clinical threshold probabilities, the application of this nomogram in clinical practice yields net clinical benefits.

## Discussion

CAR-T cell therapy has demonstrated remarkable efficacy in the treatment of R/R MM. Despite its high response rates, patients undergoing this therapy remain at risk of distinct treatment-related adverse events, among which PHT is a clinically significant concern. Previous research has linked PHT to poorer prognosis and greater healthcare burdens, including the need for continuous outpatient care, transfusions, and growth factors, in addition to increased risks of infectious and hemorrhagic complications. Furthermore, PHT may preclude adequate delivery of salvage therapies in the event of relapse (24). Therefore, early prediction of PHT is critical for risk stratification and developing individualized management strategies in high-risk patients. In this study, we developed and validated a nomogram for the early prediction of PHT in patients with R/R MM after CAR-T cell therapy. Integrating baseline clinical indicators (e.g., high tumor burden, ferritin, and IFN-γ) and CONUT-assessed nutritional status, this nomogram effectively predicts PHT. Importantly, it maintained favorable accuracy in predicting PHT_Composite, despite having been developed based on the PHT_ANC definition, indicating its generalizable predictive utility.

To date, multiple studies have sought to identify risk factors for PHT following CAR-T cell immunotherapy in patients with R/R MM. For example, Nagle et al. (25) reported a positive correlation between baseline ferritin and CRP levels and the occurrence of PHT after CAR-T cell therapy. Wang et al. (26) found that baseline bone marrow tumor burden, the severity of CRS, and levels of serum biomarkers—including peak CRP, IL-10, IFN-γ, and ferritin—were associated with the incidence of PHT after CAR-T cell therapy. Li et al. (13) identified interferon-gamma (IFN-γ) and severe hematologic toxicity following lymphodepleting chemotherapy as independent risk factors for PHT after CAR-T cell therapy. Furthermore, Rejeski et al. (14) developed the CAR-HEMATOTOX model to predict hematologic toxicity after CAR-T therapy, which incorporates baseline hematopoietic reserve (PLT, ANC, HB) and inflammatory markers (CRP, ferritin). In our study, an analysis of baseline clinical characteristics that may influence the occurrence of PHT confirmed that high tumor burden, ferritin levels, and IFN-γ are independent risk factors for PHT_ANC after CAR-T therapy. These findings are consistent with previous studies. High tumor burden can lead to excessive expansion and activation of CAR-T cells *in vivo*, resulting in massive tumor lysis and triggering severe CRS. Such intense inflammatory responses may directly damage the bone marrow microenvironment and suppress hematopoietic stem cell (HSC) function, thereby establishing a critical foundation for PHT development (27, 28). Meanwhile, ferritin, as a sensitive indicator of systemic inflammation, reflects the severity of the patient's inflammatory state. Additionally, studies suggest that IFN-γ may play a central role in the pathogenesis of PHT. IFN-γ can activate quiescent hematopoietic stem cells in response to chronic infections, potentially leading to their exhaustion—a mechanism that shares phenotypic parallels with aplastic anemia (29, 30).

The CONUT score, an immune-nutritional indicator derived from serum albumin level, peripheral lymphocyte count, and total cholesterol, serves as a screening tool for early detection of malnutrition (31). Recent studies have confirmed that a higher CONUT score is significantly associated with poorer overall survival in MM patients and can be utilized for prognostic prediction (19, 32). A study by Özkan et al. (33) further demonstrated that CONUT is an effective predictive tool for assessing early post-transplant complications in R/R MM patients, thereby guiding targeted interventions to optimize clinical management. In the present study, we identified CONUT as an independent influencing factor for PHT_ANC after CAR-T cell therapy. Notably, compared to the CAR-HEMATOTOX model developed by Rejeski et al., the nomogram constructed based on CONUT appeared to exhibit stronger discriminatory ability (AUC: 0.815 vs. 0.706; *p* < 0.001). Furthermore, when applied to predict the occurrence of PHT_Composite in R/R MM patients after CAR-T cell therapy, the nomogram achieved an AUC of 0.821, with a *p*-value of 0.417, indicating that it also demonstrates excellent discriminative capability for predicting PHT_Composite.

This study has several limitations. First, as a single-center retrospective analysis, selection bias is unavoidable. The nomogram was validated only through an internal random split and has not been tested in independent multicenter cohorts; this internal validation approach carries a risk of overfitting, which further limits the model's generalizability. Second, patients received CAR-T products with different targets. Although subgroup analysis was attempted, the limited sample size within each subgroup precluded robust comparisons. Moreover, although CONUT is commonly interpreted as a nutritional index, its components (albumin, cholesterol, and lymphocyte count) may also be influenced by systemic inflammation and disease activity; therefore, we could not fully disentangle “nutritional reserve” from “inflammatory burden” using CONUT alone, and future studies should consider integrating dedicated inflammatory markers and standardized nutritional assessments. Finally, while we used PHT as a clinically relevant endpoint, downstream clinical consequences of PHT (e.g., documented infections, transfusion burden, hospitalization duration, and survival impact within this cohort) could not be systematically evaluated because these data were not consistently available in the retrospective records. Additionally, optimal early intervention strategies for high-risk patients remain undefined. These issues warrant further investigation in prospective studies.

## Conclusions

In summary, this study developed a nomogram based on baseline clinical indicators (e.g., high tumor burden, ferritin, and IFN-γ) and CONUT-assessed nutritional status to predict the occurrence of PHT in R/R MM patients after CAR-T cell therapy. Compared with the CAR-HEMATOTOX model, our nomogram demonstrated superior predictive performance. It shows potential for early identification of R/R MM patients at high risk for PHT after CAR-T cell therapy, paving the way for individualized and scientifically grounded patient management strategies.

## Data Availability

The raw data supporting the conclusions of this article will be made available by the authors, without undue reservation.
