# Supplementary material for: Construction and validation of a nutritional status (CONUT)-based nomogram for predicting prolonged hematological toxicity in relapsed/refractory multiple myeloma after CAR-T cell therapy
Source: Front Nutr. 2026 Feb 23;13:1729151. doi: 10.3389/fnut.2026.1729151 (PMC12967921; doi:10.3389/fnut.2026.1729151)
Supplement: Supplementary file 1 [file Table_1.docx]

**Table S1** Variables of Controlling Nutritional Status score

| **Variables** | **Values** | **Score** |
| --- | --- | --- |
| Albumin (g/L) | ≥ 35 | 0 |
|  | 30-34 | 2 |
|  | 25-29 | 4 |
|  | < 25 | 6 |
| Total lymphocyte count (10^9^/L) | ≥ 1.6 | 0 |
|  | 1.2-1.599 | 1 |
|  | 0.8-1.199 | 2 |
|  | < 0.8 | 3 |
| Total cholesterol (mmol/L) | ≥ 4.65 | 0 |
|  | 3.62-4.64 | 1 |
|  | 2.58-3.61 | 2 |
|  | < 2.58 | 3 |
| normal 0–1; mild 2–4; moderate 5–8; severe 9–12. | | |
